# Evaluation of three neonicotinoid insecticides against the common pistachio psylla, *Agonoscena pistaciae* , and its natural enemies

**DOI:** 10.1093/jis/14.1.35

**Published:** 2014-01-01

**Authors:** Najmeh Amirzade, Hamzeh Izadi, Mohammad Amin Jalali, Hadi Zohdi

**Affiliations:** 1 Department of Plant Protection, Vali-e-Asr University of Rafsanjan, Iran; 2 Plant Pests and Diseases Research Center, Kerman, Iran

**Keywords:** *Adalia bipunctata*, *Coccinella undecimpunctata aegyptica*, Neonicotinoid

## Abstract

The common pistachio psylla, *Agonoscena pistaciae* Burckhardt and Lauterer (Hemiptera: Psyllidae), is a key pest in pistachio orchards in Iran. Chemical control is a common method to manage this pest. Compatibility between natural enemies and pesticides is a primary concern in programs of integrated pest management of the psyllid pest. In this research, susceptibility of fifth instar nymphs of *Ag. pistaciae* and fourth instar larvae of the two most common predators of this pest, *Adalia bipunctata* L. (Coleoptera: Coccinellidae) and *Coccinella undecimpunctata aegyptiaca* Reiche, to acetamiprid, thiamethoxam, and imidacloprid was investigated. Probit analysis of concentration-mortality data was conducted to estimate the LC50 value. The results showed that thiamethoxam with an LC50 value of 56.35 mg a.i./L was more toxic to fifth instar nymphs of *Ag. pistaciae* in comparison to acetamiprid (60.75 mg a.i/L) and imidacloprid (138.21 mg a.i/L) *.* Imidacloprid with an LC50 value of 218.89 mg a.i/L compared to acetamiprid (222.65 mg a.i/L) and thiamethoxam (232.37 mg a.i/L) had more lethal effects on fourth instar larvae of *Ad. bipunctata.* However, on the fourth instar larvae of *C. undecimpunctata aegyptica* , acetamiprid with an LC50 value of 263.44 mg a.i/L was more toxic than thiamethoxam (296.62 mg a.i/L) and imidacloprid (447.82 mg a.i/L). The laboratory findings showed that the three tested insecticides were more toxic to the common pistachio psylla than to its natural predators. Thiamethoxam was the most toxic against *Ag. pistaciae* . However, its toxicity to the predators was lower than imidacloprid and acetamiprid. This result suggests that thiamethoxam is the best insecticide for control of *Ag. pistaciae* in combination with predatory lady beetles.

## Introduction

The common pistachio psylla, *Agonoscena pistaciae* Burckhardt and Lauterer (Hemiptera: Psyllidae), is one of the most important pests of pistachio trees in Iran. This species is widely distributed throughout the pistachio growing regions of Iran, especially in Rafsanjan, the main pistachio production area in Iran. In autumn, the adult insects enter diapause under the loose bark on the trunks of pistachio trees and in soil and under leaves, where they overwinter. This pest has a multivoltine life cycle throughout Iran ( Mehrnejad, 2003 ). Chemical control is a common method in management of this pest. However, continued use of chemicals has made *Ag. pistaciae* resistant to several of the insecticides currently in use. Additionally, some of these pesticides adversely affect biological control agents used to control the psyllid, resulting in subsequent pest resurgence ( Mehrnejad 2003 ). Beneficial arthropods often exhibit greater susceptibility to persistent insecticides than their host or prey ( Croft 1990 ; Ruberson et al. 1998 ). This is caused by a variety of factors, including their active searching behavior, lower detoxification capacity, lower genetic variation, and food limitation ( Tabashnik and Johnson 1999 ). *Adalia bipunctata* L. (Coleoptera: Coccinellidae) and *Coccinella undecimpunctata aegyptiaca* Reiche are the two most notable ladybird predators of *Ag. pistaciae* in the pistachio orchards of Rafsanjan. Evidence from laboratory and field trials has demonstrated that *Ag. pistaciae* fully supports development and reproduction of these two predatory beetles ( Jalali 2001 ).

Neonicotinoids are compounds with systemic, translaminar, and contact activity ( Elbert et al. 1991 ). They have a mode of action similar to that of nicotine, acting as an agonist of the nicotinic acetylcholine receptor in the insect’s postsynaptic membrane ( Schroeder and Flattum 1984 ).

Acetamiprid, thiamethoxam, and imidacloprid are fast-acting neonicotinoid insecticides in the chloronicotinyl nitroguanidine chemical family (Kramarz and Stark 2003) and act on the insect nervous system by attaching to the acetylcholine binding sites, called nicotinergic receptors, on the receiving nerve cells. This mode of action prevents transmission of information at those binding sites, leading to lasting impairment of the nervous system and eventual death ( Matsuda et al. 2001 ; Millar and Denholm 2007 ). Because of the systemic mode of action and low toxicity to humans, neonicotinoids have become popular insecticides worldwide for control of sap-sucking insects, some chewing insects (including termites), soil insects, and fleas on pets ( Matsuda et al. 2001 ; Nauen and Denholm 2005 ; Herbert et al. 2008).

Sole use of biological control may not always be sufficient to manage insect pest populations, and appropriate insecticide treatments may be needed ( Hassan and Van de Veire 2004 ). Therefore, it is necessary to identify insecticides for use in integrated pest management programs to effectively control *Ag. pistaciae* with minimal impact on associated biological control agents. In the current study, the toxicity of three neonicotinoid insecticides to fifth instar nymphs of *Ag. pistaciae* , and their compatibility with fourth instar *Ad. bipunctata* and *C. undecimpunctata aegyptiaca* , was assessed. The fourth instar of each predator was selected because of their superior predation capacity relative to that of younger instars ( Jalali 2001 ), and hence their greater expected contribution to biological control. Tested compounds are routinely used against *Ag. pistaciae* on cultivated pistachio trees in Rafsanjan, where the two coccinellid species studied may be additional assets in integrated pest management programs.

## Materials and Methods

### Rearing of the test insects

*Ag. pistaciae* was reared on pistachio shrubs in ventilated aluminum rearing cages (50 ×60 ×70 cm) in a greenhouse under controlled conditions at 26 ± 2°C, 60–70% RH, and a photoperiod of 16:8 L:D. Adults of the predatory beetles were collected from pistachio gardens in Rafsanjan. Lady beetles were reared in ventilated plastic boxes (20 ×25 ×10 cm) at 26 ± 2ºC, 65 ± 5% RH, with a photoperiod of 16:8 L:D. They were provided with *Ag. pistaciae* as food and were left for at least 3 weeks to adapt to the laboratory conditions.

### Insecticides

Commercial formulations of imidacloprid (Confidor, 0.35% suspension concentration, Bayer CropScience, www.cropscience.bayer.com ), acetamiprid (Aventis, 20% suspension powder, Bayer CropScience), and thiamethoxam (Actara, 25% wetable granule, Syngenta, www.syngenta.com ) were used. Based on preliminary tests, graded concentrations of pesticides (20, 40, 80, 120, and 160 mg a.i./L for acetamiprid; 25, 50, 75, 100, and 125 mg a.i./L for thiamethoxam; 53, 70, 105, 147, and 210 mg a.i./L for imidacloprid) were prepared with distilled water.

### Bioassay of *Ag. pistaciae*

To study the susceptibility of the fifth instar nymphs of *Ag. pistaciae* to acetamiprid, thiamethoxam, and imidacloprid, one-day-old fifth instar nymphs were dipped in different concentrations of insecticides for 2 sec. Treated nymphs were allowed to air dry and then placed on the pistachio leaves and assayed under controlled conditions. The experiments were carried out with 3 replications, each consisting of 20 nymphs. Distilled water was used as the control. Treated nymphs were maintained in a climate chamber and reared on fresh leaves. Mortality counts were made after 24 hr.

### Bioassays of *Ad. bipunctata* and *C. undecimpunctata aegyptica*

Susceptibility of the fourth instar larvae of the predators *Ad. bipunctata* and *C. undecimpunctata aegyptica* to different concentrations of acetamiprid, thiamethoxam, and imidacloprid was investigated by a topical application method using an automatic micro-syringe pump (Stoelting, www.stoelting.com ). Thirty insects were assayed per concentration (10 larvae per replicate, 3 replications). Distilled water was used as a control. Treated larvae were provided with *Ag. pistaciae* and maintained in a growth chamber at 26 ± 2ºC, 65 ± 5% RH, with a photoperiod of 16:8 L:D. Survival was checked after 24 hr.

### Experimental design and data analysis

The factorial experiment was in a randomized complete block design, with two factors: (1): treatments: 3 insecticides and the control, each consisting of 3 replications and 5 concentrations; and (2) 3 insects, each consisting of 20 nymphs. Data were analyzed with Minitab 14 ( Minitab 2005 , www.minitab.com ) software followed by MSTAT-C to compare effects among treatments. The results were expressed as percent of larval mortality. Probit analysis was used for estimation of LC50 by POLO-PC 2002 software ( Robertson et al. 2007 ; www.polo-pc-2002.software.informer.com ).

## Results

In this study, the effects of 3 neonicotinoid insecticides, i.e. acetamiprid, thiamethoxam, and imidacloprid, were investigated for their effect on the mortality of fifth instar nymphs of *Ag. pistaciae* and fourth instar larvae of its natural predators, lady beetles *Ad. bipunctata* and *C. undecimpunctata aegyptica* . In acetamiprid, thiamethoxam, and imidacloprid treatments, mortality of *Ag. pistaciae* and its predators increased as the concentration of the insecticides increased. Mortality was proportional to pesticide concentration ( Tables 1–3 ). In the bioassay of the tested insecticides against *Ag. pistaciae* , there were significant differences in mortality between the insecticides and the control (F6, 35 = 16.584, *p* < 0.05). In the bioassay of the tested insecticides against fourth instar larvae of *Ad. bipunctata* and *C. undecimpunctata aegyptica* , there were significant differences in mortality between the insecticides and the control ( *F* 3, 8 = 9.032, *p* < 0.05).

**Table 1.**
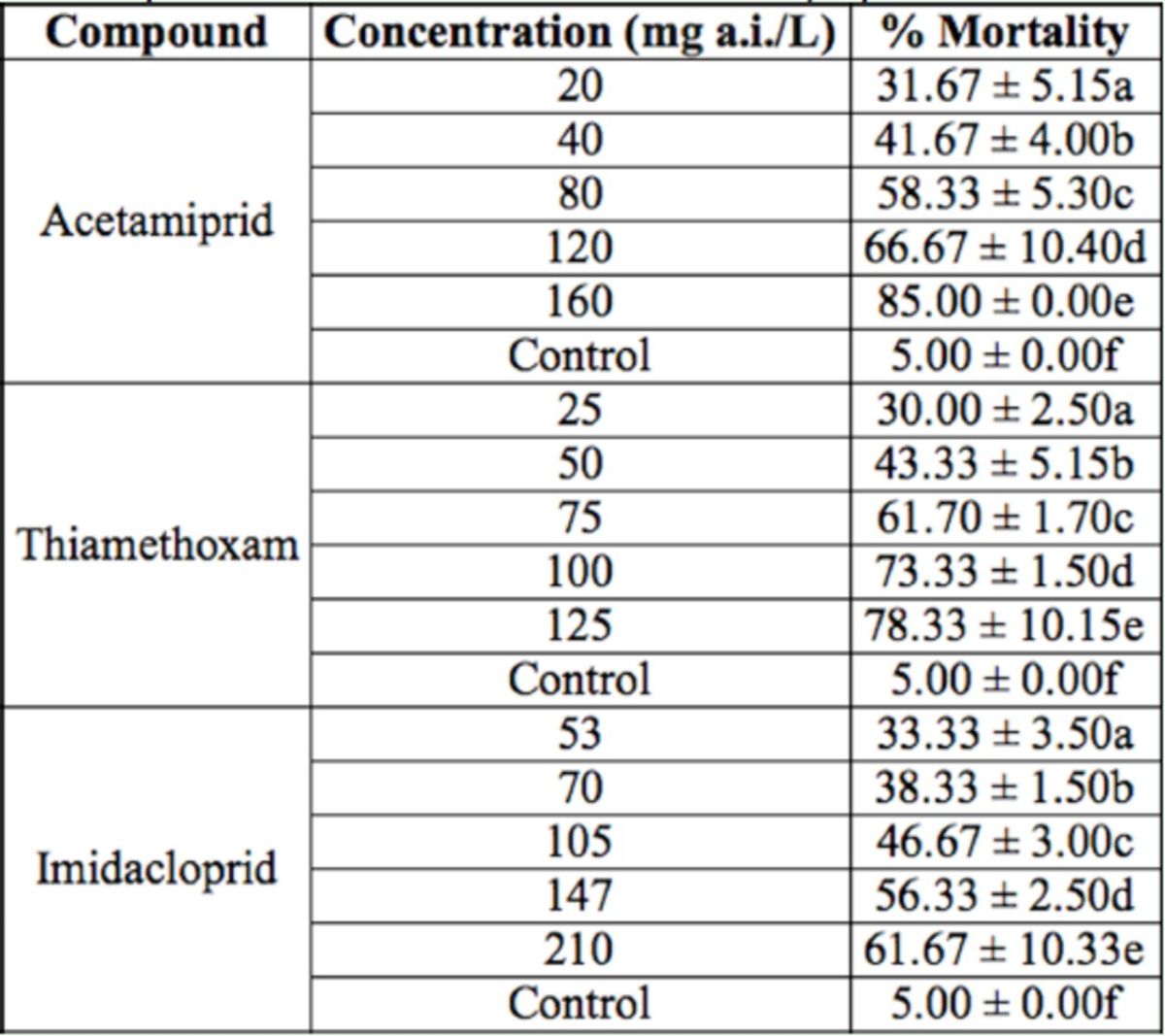
Mortality of *Agonoscena pistaciae* fifth instar nymphs treated with acetamiprid, thiamethoxam, and imidacloprid. For each insecticide, means within a column followed by the same letter are not significantly different ( *p* > 0.01). The experiments were replicated 3 times with 20 fifth instar nymphs.

**Table 2.**
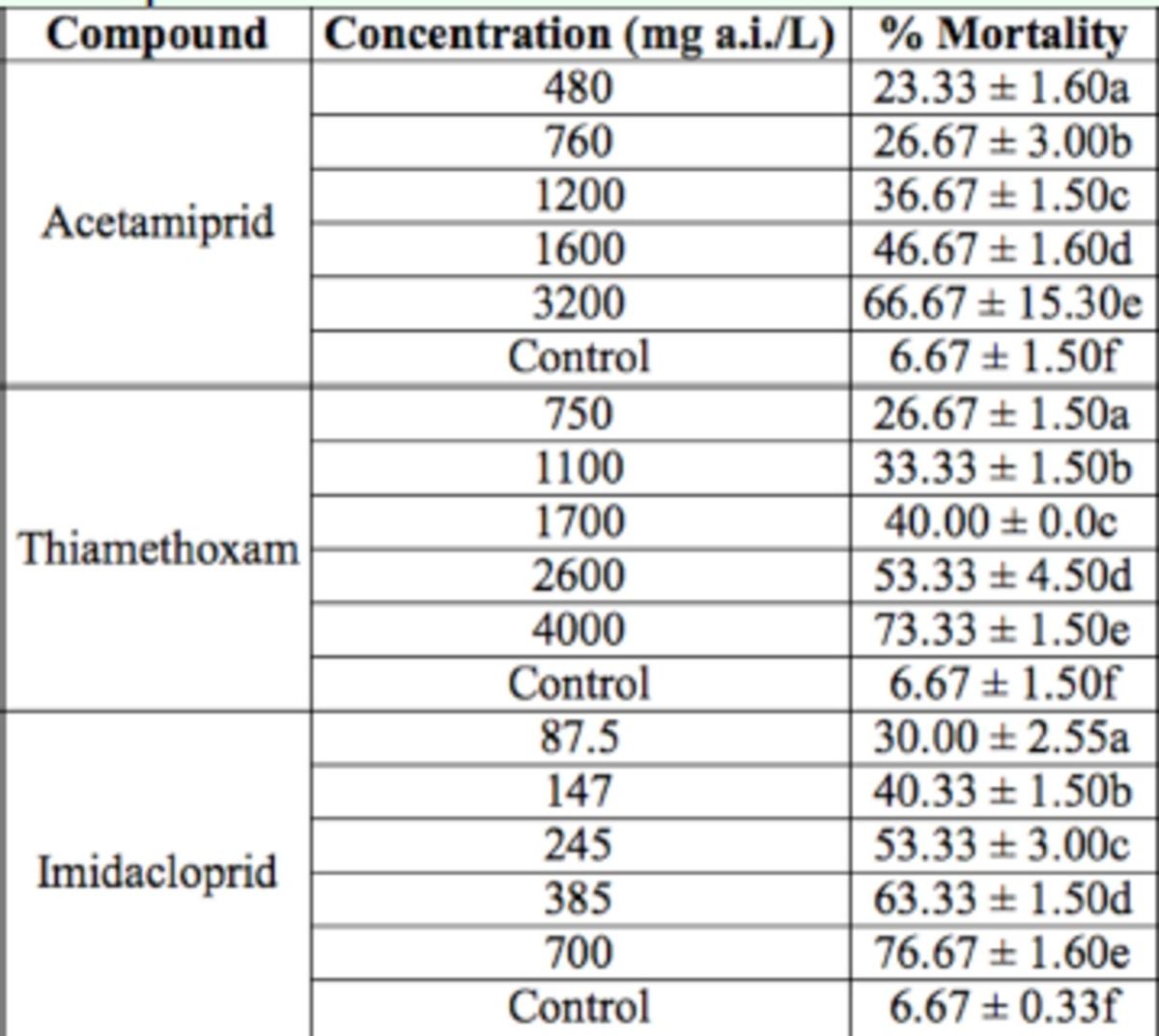
Mortality of *Adalia bipunctata* fourth instar larvae treated with acetamiprid, thiamethoxam, and imidacloprid. For each insecticide, means within a column followed by the same letter are not significantly different ( *p* > 0.01). The experiments were replicated 3 times with 20 fourth instar larvae.

**Table 3.**
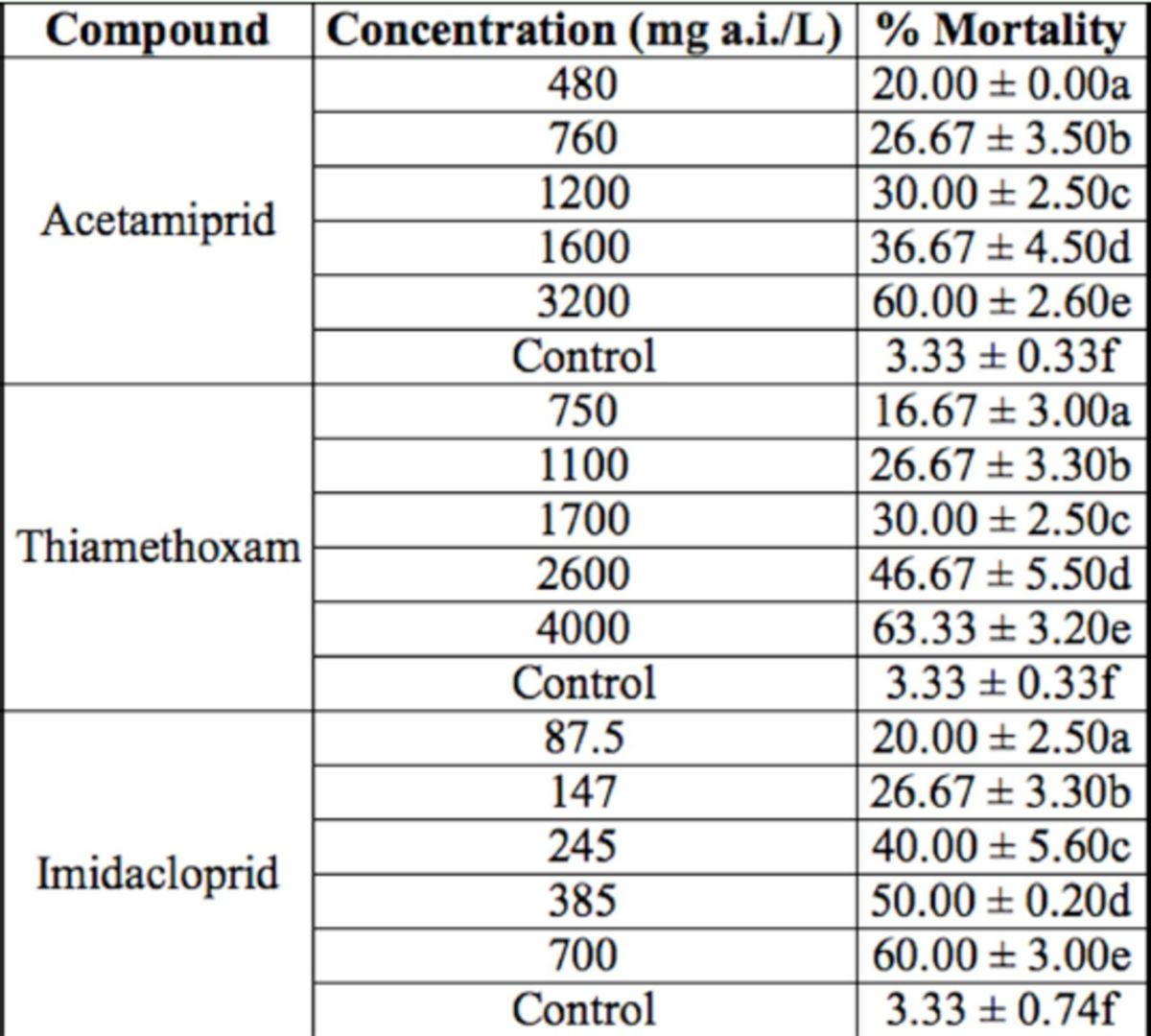
Mortality of *Coccinella undecimpunctata aegyptica* fourth instar larvae treated with acetamiprid, thiamethoxam, and imidacloprid. For each insecticide, means within a column followed by the same letter are not significantly different ( *p* > 0.01). The experiments were replicated 3 times with 20 fourth instar larvae.

Probit analysis revealed that thiamethoxam (LC50 = 56.35 mg a.i/L) was more toxic to *Ag. pistaciae* than acetamiprid (LC50= 60.70 mg a.i/L) and imidacloprid (LC50= 138.21 mg a.i/L) ( Table 4 ). The toxicity of 3 different neonicotinoids to the fifth instar nymphs of *Ag. pistaciae* was in the following order: thiamethoxam > acetamiprid > imidacloprid.

**Table 4.**
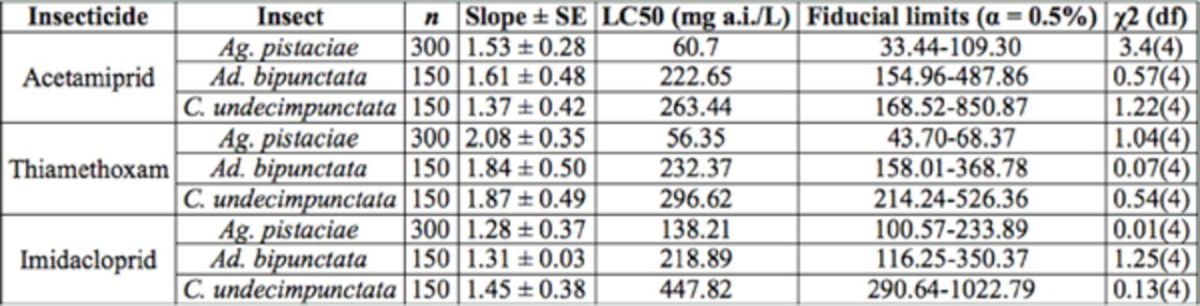
Evaluation of imidacloprid, acetamiprid, and thiamethoxam on the mortality of fifth instar nymphs of *Agonoscena pistaciae* and fourth instar larvae of *Adalia bipunctata* and *Coccinella undecimpunctata aegyptica* . χ2 = chisquare; df = degree of freedom, n = number of treated larvae.

In evaluation of acetamiprid against *Ag. pistaciae* and *Ad. bipunctata* and *C. undecimpunctata aegyptica* , Chisquare values (3.4, 0.57, and 1.22, respectively) indicated that this insecticide had the best fit to the *Ad. bipunctata* data (Chisquare for this species was 5.96-fold less than for *Ag. pistaciae* and 2.14-fold less than for *C. undecimpunctata aegyptica* ). In evaluation of thiamethoxam, Chisquare values (1.04, 0.07, and 0.54 for *Ag. pistaciae* , *Ad. bipunctata* , and *C. undecimpunctata aegyptica* , respectively) indicated that this insecticide had the best fit to the *Ad. bipunctata* data (Chisquare for this species was 14.85-fold less than for *Ag. pistaciae* and 7.71-fold less than for *C. undecimpunctata aegyptica* ). In evaluation of imidacloprid, Chisquare values (0.01, 1.25, and 0.13 for *Ag. pistaciae* , *Ad. bipunctata* , and *C. undecimpunctata aegyptica* , respectively) indicated that this insecticide had the best fit to the *Ag. pistaciae* data (Chisquare for this species was 125-fold less than for *Ad. bipunctata* and 13-fold less than for *C. undecimpunctata aegyptica* ). The degrees of freedom of the insecticides were fixed for all the tested species ( Table 4 ).

The LC50 values comparing the LC50 ratio (2.45) and the lower and upper 95% confidence limits (0.15–0.8) showed that there was a significant difference between the LC50 values of imidacloprid (138.21 mg a.i./L) and thiamethoxam (56.35 mg a.i./L) ( Table 4 ). In the comparison between imidacloprid and acetamiprid, the LC50 ratio (2.28) with the lower and upper 95% confidence limits (0.11–0.67) showed that there was a significant difference between the LC50 values of imidacloprid (138.21 mg a.i./L) and acetamiprid (60.70 mg a.i./L) ( Table 4 ). In the comparison between acetamiprid and thiamethoxam, the LC50 ratio (1.07) with the lower and upper 95% confidence limits (-1.73–1.75) showed that there was not a significant difference between the LC50 values of acetamiprid (60.70 mg a.i./L) and thiamethoxam (56.35 mg a.i./L) ( Table 4 ).

Probit analysis data revealed that imidacloprid (LC50 = 218.89 mg a.i/L) was more toxic to *Ad. bipunctata* than acetamiprid (LC50 = 222.65 mg a.i/L) and thiamethoxam (LC50 = 232.37 mg a.i/L). The toxicity of the 3 different neonicotinoids to the fourth instar larvae of *Ad. bipunctata* was in the following order: imidacloprid > acetamiprid > thiamethoxam ( Table 4 ).

The LC50 values comparing the LC50 ratio (1.04) and the lower and upper 95% confidence limits (-0.26–0.34) showed that there was a significant difference between the LC50 values of acetamiprid (222.65 mg a.i/L) and thiamethoxam (232.37 mg a.i/L) ( Table 4 ). In the comparison between imidacloprid and acetamiprid, the LC50 ratio (1.17) with the lower and upper 95% confidence limits (-0.32–0.36) showed that there was a significant difference between the LC50 values of imidacloprid (218.89 mg a.i/L) and acetamiprid (222.65 mg a.i/L) ( Table 4 ). In the comparison between imidacloprid and thiamethoxam, the LC50 ratio (1.06) with the lower and upper 95% confidence limits (-0.18–0.22) showed that there was a significant difference between the LC50 values of thiamethoxam (232.37 mg a.i/L) and imidacloprid (218.89 mg a.i/L) ( Table 4 ).

Probit analysis revealed that acetamiprid (LC50 = 263.44 mg a.i/L) was more toxic to *C. undecimpunctata aegyptica* than thiamethoxam (LC50 = 296.63 mg a.i/L) and imidacloprid (LC50 = 447.82 mg a.i/L) ( Table 4 ). The toxicity of the 3 different neonicotinoids to the fourth instar larvae of *C. undecimpunctata aegyptica* was in the following order: acetamiprid > thiamethoxam > imidacloprid ( Table 4 ).

The LC50 values comparing the LC50 ratio (1.13) and the lower and upper 95% confidence limits (-0.35–0.45) showed that there was a significant difference between the LC50 values of acetamiprid (263.44 mg a.i/L) and thiamethoxam (296.62.00 mg a.i/L) ( Table 4 ). In the comparison between imidacloprid and acetamiprid, the LC50 ratio (1.70) with the lower and upper 95% confidence limits (0.13– 0.33) showed that there was a significant difference between the LC50 values of imidacloprid (447.82 mg a.i/L) and acetamiprid (263.44 mg a.i/L) ( Table 4 ). In the comparison between imidacloprid and thiamethoxam, the LC50 ratio (1.51) with the lower and upper 95% confidence limits (-0.22–0.58) showed that there was a significant difference between the LC50 values of imidacloprid (447.82 mg a.i/L) and thiamethoxam (296.62 mg a.i/L) ( Table 4 ).

## Discussion

The rate of change of mortality in relation to a unit change in concentration is expressed by the slope of a line, which in turn expresses the variability in susceptibility of the test population. A steep line means a population has a small variation in susceptibility, whereas a flat line means a population varies widely in susceptibility. In the bioassay of *Ag. pistaciae* , the slope of the dose-response line of thiamethoxam was steep and the difference between the highest and lowest concentrations was low. That means the population was homogeneous in susceptibility, and with a fairly small increase in insecticide concentration the mortality would increase considerably. This finding necessitates more careful use of this neonicotinoid in the field to prevent exerting a high selection pressure that could eliminate the susceptible individuals and lead to selection of resistant individuals. The slopes of the dose-response lines of imidacloprid and acetamiprid were flat, and the difference between the highest and lowest concentrations was high. That means the mortality would increase considerably with a fairly large increase in insecticide concentration ( Robertson et al. 2007 ).

The results indicated high toxicity of imidacloprid, acetamiprid, and thiamethoxam against *Ad. bipunctata.* The slopes of the dose-response lines of the tested insecticides were flat, and the differences between the highest and lowest concentrations were high, which means the mortality would increase considerably with a fairly large increase in insecticide concentration. This finding is in agreement with the results of Torres and Ruberson (2004) , Bozsik (2006) , and Jalali et al. (2009) . In addition, Michaud (2002) showed that imidacloprid was toxic to larvae of *Harmonia axyridis* and *Cycloneda sanguinea* using a similar method of exposure. Dong-Soon et al.(2006) found high toxicity of acetamiprid against *Deraeocoris brevis.*

The sensitivity of *C. undecimpunctata aegyptica* to imidacloprid was found to be quite different from the sensitivity of *Ad. bipunctata* . *Ad. bipunctata* was 2 times more sensitive than *C. undecimpunctata* , but in general the sensitivity of *Ad. bipunctata* to all tested insecticides was more than the sensitivity of *C. undecimpunctata aegyptica* .

In conclusion, the findings indicated that the 3 tested insecticides were more toxic to *Ag. pistaciae* than to its natural predators the lady beetles. Thiamethoxam was the most toxic insecticide against *Ag. pistaciae* . However, its toxicity to predatory lady beetles was lower than imidacloprid and acetamiprid. So, it could be concluded from the results that thiamethoxam is the best insecticide for control of *Ag. pistaciae* in combination with predatory lady beetles.

## References

[R1] Bozsik A . 2006 . Susceptibility of adult *Coccinella septempunctata* (Coleoptera: Coccinellidae) to insecticides with different modes of action . Pest Management Science 62 : 651 – 654 . 16649191 10.1002/ps.1221

[R2] Croft BA . 1990 . Arthropod Biological Control Agents and Pesticides . Wiley and Sons .

[R3] Dong-Soon K Deborah JB Helmut R . 2006 . Lethal and sublethal effects of abamectin, spinosad, methoxyfenozide and acetamiprid on the predaceous plant bug *Deraeocoris brevis* in the laboratory . Biocontrol 51 : 465 – 484 .

[R4] Elbert A Becker B Hartwig J Erdelen C . 1991 . Imidacloprid–a new systemic insecticide . Pflanzenschutz Nachrichten Bayer 44 : 113 – 136 .

[R5] Hassan SA Van de Veire M. 2004 . Compatibility of pesticides with biological control agents. In: Heinz KM, Parella MP, van Driesche RM, Editors . Biocontrol in protected culture . pp. 129 – 147 . Chicago Review Press .

[R6] Herbert KS Hoffmann AA Powell KS . 2008 . Assaying the potential benefits of thiamethoxam and imidacloprid for phylloxera suppression and improvements to grapevine vigour . Crop Protection 27 : 1229 – 1236 .

[R7] Jalali MA . 2001 . Study of food consumption in predatory beetles (Col.: Coccinellidae) of the common pistachio psyllid, Agonoscena pistaciae in Rafsanjan, and compiling a life table in the controlled condition . M.Sc. Thesis, College of Agriculture, the University of Shiraz , Iran .

[R8] Jalali MA Vanleeuwen T Tirry L Declercq P . 2009 . Toxicity of selected insecticides to the two-spot ladybird *Adalia bipunctata* . Phytoparasitica 37 : 323 – 326 .

[R9] Kramarza P Stark JD . 2003 . Population level effects of cadmium and the insecticide imidacloprid to the parasitoid, *Aphidius ervi* after exposure through its host, the pea aphid, *Acyrthosiphon pisum* (Harris) . Biological Control 27 : 310 – 314 .

[R10] Matsuda K Buckingham SD Kleier D . 2001 . Neonicotinoids: insecticides acting on insect nicotinic acetylcholine receptors . Trends in Pharmacological Sciences 22 : 573 – 579 . 11698101 10.1016/s0165-6147(00)01820-4

[R11] Mehrnejad MR . 2003 . Pistachio psylla and other major psyllids of Iran . Publication of the Agricultural Research and Education Organization , Tehran, Iran .

[R12] Mehrnejad MR Jalali MA Mirzaei R . 2011 . Abundance and biological parameters of psyllophagous coccinellids in pistachio orchards . Journal of Applied Entomology 135 : 673 – 681 .

[R13] Michaud JP . 2002 . Relative toxicity of six insecticides to *Cycloneda sanguinea* and *Harmonia axyridis* (Coleoptera: Coccinellidae) . Journal of Entomological Science 37 : 82 – 93 .

[R14] Millar NS Denholm I . 2007 . Nicotinic acetylcholine receptors: targets for commercially important insecticides . Invertebrate Neuroscience 7 : 53 – 66 . 17216290 10.1007/s10158-006-0040-0

[R15] Minitab . 2005 . Meet MINITAB, Release 14, for Windows . Minitab Inc .

[R16] MSTAT Development Team . 1983 . MSTAT-C, A microcomputer program for the design, management and analysis of agronomic research experiment . Michigan State University .

[R17] Nauen R Denholm I . 2005 . Resistance of insect pests to neonicotinoid insecticides: current status and future prospects . Archives of Insect Biochemistry 58 : 200 – 215 . 10.1002/arch.20043 15756698

[R18] Robertson JL Russell RM Preisler HK Savin NE . 2007 . Pesticide Bioassays with Arthropods . CRC Press .

[R19] Ruberson J.R. Nemoto H Hirose Y . 1998 . Pesticides and conservation of natural enemies. In: Barbosa P, Editor . pp. 207 – 220 . Conservation Biological Control . Academic Press .

[R20] Schroeder ME Flattum RF . 1984 . Mode of action and neurotoxic properties of the nitromethylene heterocyclic insecticides . Pesticide Biochemistry and Physiology 22 : 148 – 160 .

[R21] Tabashnik BE Johnson MW . 1999 . Evolution of pesticide resistance in natural enemies. In: Bellows TS, Fisher TW, Editors . Handbook of Biological Control . pp. 673 – 689 . Academic Press .

[R22] Torres JB Ruberson J.R. . 2004 . Toxicity of thiamethoxam and imidacloprid to *Podisus nigrispinus* (Dallas) (Heteroptera: Pentatomidae) nymphs associated to aphid and whitefly control in cotton . Neotropical Entomology 33 : 99 – 106 .

